# Human antigen R-regulated CCL20 contributes to osteolytic breast cancer bone metastasis

**DOI:** 10.1038/s41598-017-09040-4

**Published:** 2017-08-29

**Authors:** Sun Kyoung Lee, Kwang-Kyun Park, Hyun-Jeong Kim, Junhee Park, Seung Hwa Son, Ki Rim Kim, Won-Yoon Chung

**Affiliations:** 1 Department of Oral Biology, Oral Cancer Research Institute, BK21 PLUS Project, Yonsei University College of Dentistry, Seoul, 03722 Republic of Korea; 2 Department of Applied Life Science, The Graduate School, Yonsei University, Seoul, 03722 Republic of Korea; 3 Department of Dentistry, The Graduate School, Yonsei University, Seoul, 03722 Republic of Korea; 4 Department of Dental Hygiene, Gangdong College, Icheon, 27600 Republic of Korea; 5 Department of Dental Hygiene, College of Science and Engineering, Kyungpook National University, Sangju, 37224 Republic of Korea

## Abstract

Breast cancer mainly spreads to bone, causing decreased survival of patient. Human antigen R (HuR) and chemokines are important molecules associated with mRNA stability and cell-cell interaction in cancer biology. Here, HuR knockdown inhibited bone metastasis and osteolysis of metastatic breast cancer cells in mice and HuR expression promoted the metastatic ability of cancer cells via CCL20 and GM-CSF. In contrast with the findings for *GM-CSF*, *ELAVL1* and *CCL20* expressions were markedly increased in breast tumor tissues and *ELAVL1* expression showed a strong positive correlation with *CCL20* expression in breast cancer subtypes, particularly the basal-like subtype. Metastasis-free survival and overall survival were decreased in the breast cancer patients with high *CCL20* expression. We further confirmed the role of CCL20 in breast cancer bone metastasis. Intraperitoneal administration of anti-CCL20 antibodies inhibited osteolytic breast cancer bone metastasis in mice. Treatment with CCL20 noticeably promoted cell invasion and the secretion of MMP-2/9 in the basal-like triple-negative breast cancer cell lines, not the luminal. Moreover, CCL20 elevated the receptor activator of nuclear factors kappa-B ligand/osteoprotegerin ratio in breast cancer and osteoblastic cells and mediated the crosstalk between these cells. Collectively, HuR-regulated CCL20 may be an attractive therapeutic target for breast cancer bone metastasis.

## Introduction

Breast cancer cells favor osteolytic bone metastasis with significant bone resorption. This leads to the development of severe skeletal-related events (SREs), including bone pain, pathological fractures, nerve compression syndromes, and hypercalcemia, in approximately 70% of breast cancer patients, causing decreased survival and poor quality of life^1^. Breast cancer-mediated osteolysis is highly affected by interactions between breast cancer metastases and bone marrow stromal cells, including osteoblasts and osteoclasts^2, 3^. Breast cancer bone metastases secrete various soluble factors^4–6^, which stimulate osteoclast-mediated bone resorption through the dysregulation of osteoblastic receptor activator of nuclear factor kappa-B ligand (RANKL) and osteoprotegerin (OPG) expression^7^. Abnormally enhanced bone resorption leads to the release of matrix-stored growth factors, which activate cancer cells^8–11^. This ‘vicious cycle’ has been recognized to accelerate the growth of bone metastases and to aggravate bone damage. Thus, controlling this cycle should greatly contribute to the inhibition and treatment of cancer-associated bone destruction. Currently, bone-modifying agents, such as bisphosphonates and denosumab, a monoclonal antibody against RANKL, are used to treat SREs caused by bone metastases. Although these treatments can inhibit interactions between cancer cells and the bone microenvironment by targeting osteoclastic activity, they do not prevent the development of bone metastasis in patients and therefore do not prolong survival^12^. For the more effective treatment of breast cancer bone metastasis, the identification of new targets is required.

Human antigen R (HuR), a member of the embryonic lethal abnormal vision (ELAV)/human (Hu) family of RNA-binding proteins, binds to 3′ untranslated regions (UTRs) of target mRNAs containing AU-rich elements (AREs) and regulates their translation by enhancing their stability^13^. High HuR expression levels have been detected in almost all types of cancer tissue^14^. Overexpression of cytoplasmic HuR has been shown to modulate cancer development and progression by enhancing the expression of growth-stimulating, proto-oncogenic, and pro-angiogenic factors in several types of cancers^15–21^. This overexpression can also promote the invasiveness and metastatic ability of cancer cells by stabilizing mRNAs encoding matrix metalloproteinase (MMP)-9, metastasis-associated protein 1, and urokinase plasminogen activator (uPA)^22, 23^. Moreover, HuR has been reported to regulate the expression of parathyroid hormone-related protein, a key osteolytic factor, in human cancer cells with bone tropism^24, 25^. However, the role of HuR in breast cancer bone metastasis remains unclear.

Chemokines are chemoattractant cytokines that bind to members of the G protein-coupled receptor family and are induced by growth factors and inflammatory stimuli. Under normal physiological conditions, complexes of chemokines and their receptors modulate leukocyte trafficking during inflammatory responses^26^. In cancer, chemokines and chemokine receptors regulate cancer cell growth, migration, invasion, and metastasis and mediate interactions between tumor cells and their microenvironments^27–30^. With regard to bone metastasis, CXC chemokine ligand 12 (CXCL12/SDF-1) and its receptor, CXCR4, participate in the development of skeletal metastasis by attracting cancer cells that express a high level of CXCR4 to bone marrow containing abundant CXCL12^31, 32^. CC chemokine ligand 2 (CCL2) exerts its pro-tumorigenic and angiogenic effects through the recruitment of tumor-associated macrophages and has been implicated in various metastatic processes, including the development of bone metastasis^33^. Additionally, CXCL8, also known as interleukin-8, directly stimulates osteoclastogenesis and bone resorption; the CXCL8 level in circulation has been associated with breast cancer bone metastasis in mice and humans^34^. Therefore, chemokines acting as cancer cell-derived osteolytic factors may be promising therapeutic targets due to their effects on the bone microenvironment, as well as on cancer cells. Here, we demonstrate that HuR-regulated CCL20 are attractive targets for breast cancer bone metastasis.

## Results

### HuR knockdown inhibits bone metastasis of breast cancer cells

**Figure 1:**
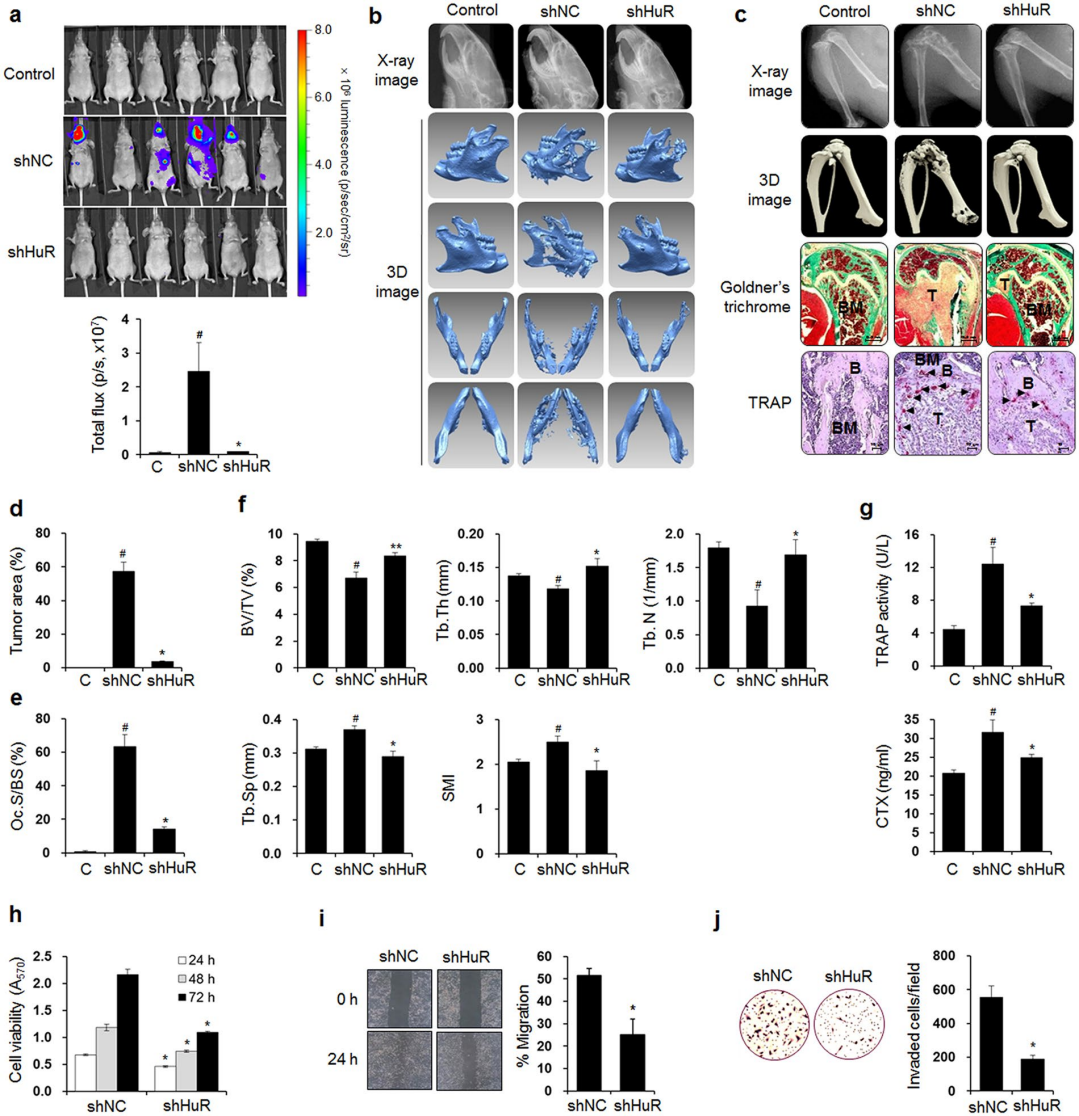
HuR knockdown prevents bone metastasis of breast cancer cells in mice. (**a**) Bioluminescence images taken 6 weeks after luciferase-transfected HuR-knockdown (shHuR) and control (shNC) breast cancer cells were inoculated into the left cardiac ventricles of nude mice (*n* = 10). (**b,c**) X-ray and 3D images of mandibles (**b**), femora, and tibiae (**c**) derived from μCT scans performed on week 6. (**c**) Goldner’s trichrome and TRAP staining of femoral tissues. Arrowheads: TRAP-positive osteoclasts; T: tumor; B: bone; BM: bone marrow. Scale bar: 0.5 mm for Goldner’s trichrome staining and 10 μm for TRAP staining. (**d,e**) Tumor areas (**d**) and osteoclast surface per bone surface (Oc.S/BS) (**e**) values for stained femoral sections. (**f**) Bone morphometric parameters BV/TV (%), Tb.Th (mm), Tb.N (1/mm), Tb.Sp (mm), and SMI of the mouse femora analyzed by μCT. (**g**) Serum levels of the bone resorption markers TRAP 5b and CTX quantified using commercial kits. The data are expressed as the mean ± standard error (s.e.m.). ^#^ *P* < 0.05 *versus* control mice without cancer cells; *P < 0.05, **P < 0.01 *versus* mice injected with shNC MDA-MB-231 cells. (**h**) Viabilities of shNC and shHuR MDA-MB-231 cells. The cells were cultured for 24, 48, and 72 h, and cell viability was assessed by MTT assay. (**i**) Cell migration. Movement of cells in scratched areas was observed under an inverted optical microscope (original magnification, × 40) and measured using ImageJ software at 0 h and 24 h. (**j**) Cell invasion. Cell invasion was measured by transwell invasion assay. Representative images of cells that invaded the lower surfaces of the filters were captured using an optical microscope (original magnification, × 200). The data are expressed as the mean ± s.e.m. *P < 0.01 *versus* shNC.

To determine whether the expression of HuR, an RNA-binding post-transcriptional regulator, is essential for breast cancer bone metastasis, we used an MDA-MB-231 basal-like/triple-negative human breast cancer cell line that has exhibited well-characterized bone tropism in animal models^35, 36^ and established luciferase-transfected HuR-knockdown (shHuR) cells and control (shNC) cells with corresponding non-specific scrambled shRNA (Supplementary Fig. 1a). The shNC and shHuR cells were inoculated into the left cardiac ventricles of nude mice, and 6 weeks later, breast cancer metastasis was detected by bioluminescence imaging. The metastasis was substantially inhibited in the shHuR cell-inoculated mice compared with the shNC cell-inoculated mice, as indicated by the bioluminescence images (Fig. 1a). X-ray and three-dimensional (3D) images derived from the micro-computed tomography (μCT) data revealed that osteolytic lesions were notably decreased in the mandibles (Fig. 1b), distal femora, and proximal tibiae (Fig. 1c) of the shHuR cell-injected mice compared with those of the shNC-inoculated mice. Goldner’s trichrome staining also showed that HuR knockdown significantly inhibited tumor growth in bone marrow (Fig. 1c,d). Bone resorption in the femora was supported by the reduced number of tartrate-resistant acid phosphatase (TRAP)-positive osteoclasts on the bone surfaces near the tumors in the shHuR cell-injected mice compared with the shNC cell-injected mice (Fig. 1c,e). In the distal femora, the bone volume over total volume (BV/TV), trabecular thickness (Tb.Th), and trabecular number (Tb.N) were reduced in the shNC cell-inoculated mice compared with the control mice, whereas trabecular separation (Tb.Sp) and the structure model index (SMI) were increased. These alterations were significantly inhibited by HuR knockdown (Fig. 1f). The serum levels of the bone resorption markers TRAP 5b and C-terminal cross-linking telopeptide of type I collagen (CTX) were increased in the shNC-cell inoculated mice, and these increases were also markedly inhibited by HuR knockdown (Fig. 1g). We confirmed that HuR knockdown inhibited tumor growth and osteolysis in a murine intratibial model of breast cancer bone metastasis (Supplementary Fig. 1b–f).

To further verify the *in vivo* inhibitory effect of HuR knockdown on breast cancer metastasis, we assessed the viability, migration, and invasion of shNC and shHuR MDA-MB-231 cells using MTT, wound-healing, and transwell invasion assays, respectively. Cell viability was decreased by 32.1%, 37.1%, and 49.5% at 24, 48, and 72 h, respectively, in the shHuR cells compared with the shNC cells (Fig. 1h). Scratch wound closure was inhibited by 50.4% (Fig. 1i) and the number of invaded cells was reduced by 66.0% following HuR knockdown (Fig. 1j). These results indicate that HuR expression in breast cancer cells is closely associated with their metastatic potential and production of osteolytic lesions.

### HuR influences breast cancer cells via CCL20 and granulocyte-macrophage colony-stimulating factor

**Figure 2:**
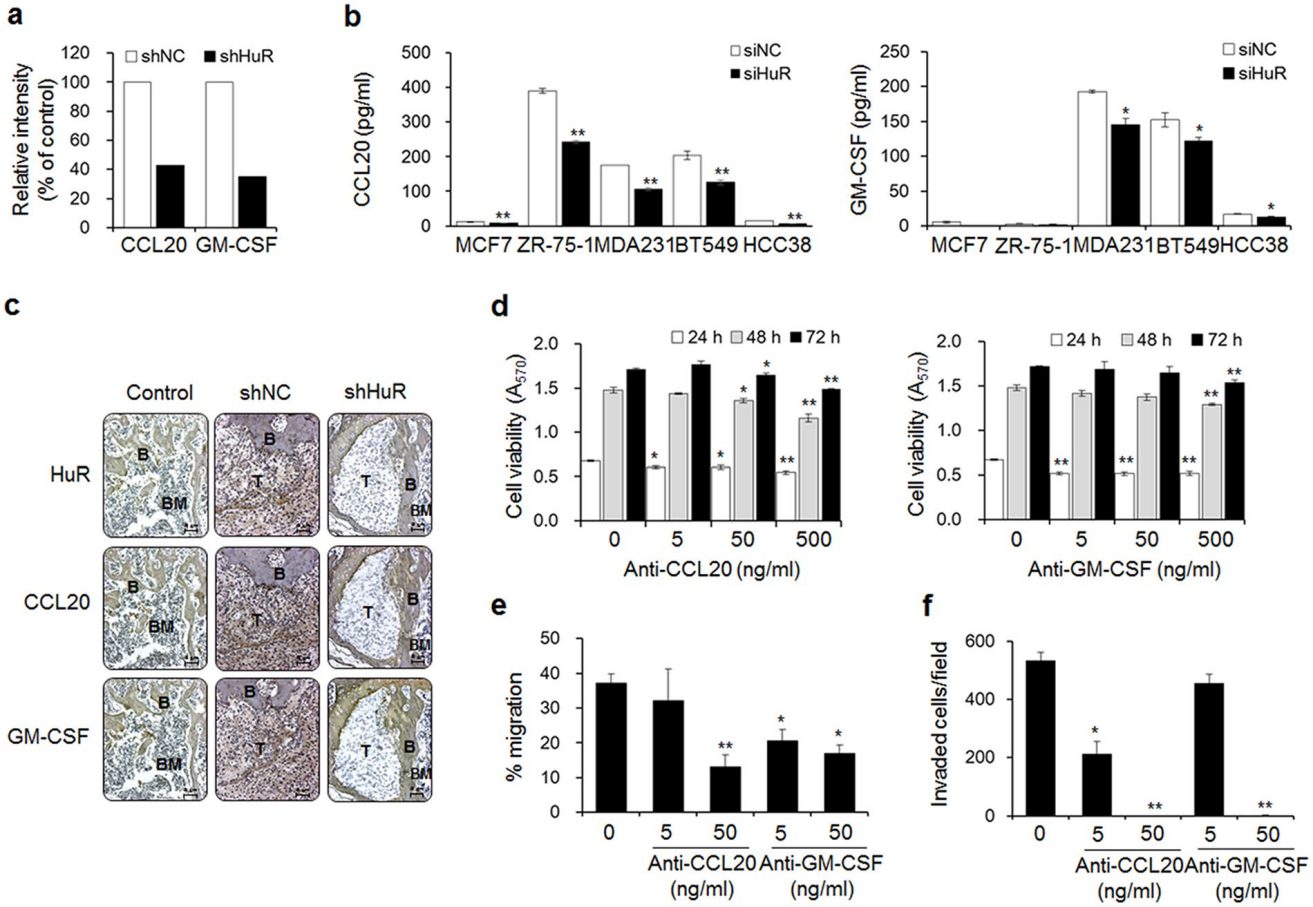
HuR improves the metastatic potential of breast cancer cells via CCL20 and GM-CSF. (**a**) The relative intensities of CCL20 and GM-CSF spots on membranes of human cytokine Ab arrays treated with conditioned media from shNC and shHuR MDA-MB-231 cells. The relative intensities of the CCL20 and GM-CSF spots were plotted as a % of the control. (**b**) The levels of secreted CCL20 and GM-CSF in multiple siNC and siHuR breast cancer cell lines. The levels of CCL20 and GM-CSF in the culture medium of each cell line were measured using commercial human CCL20 and GM-CSF ELISA kits, respectively. The data are expressed as the mean ± s.e.m. *P < 0.05, **P < 0.01 *versus* siNC. (**c**) Immunohistochemical staining of HuR, CCL20, and GM-CSF in femoral tumor tissues from mice injected with shNC or shHuR breast cancer cells, as shown in Fig. 1. T: tumor; B: bone; BM: bone marrow. Scale bar: 5 μm. (**d**) Viabilities of breast cancer cells treated with the indicated concentrations of anti-CCL20 or anti-GM-CSF antibody for 24, 48, and 72 h. Cell viability was determined by MTT assay. (**e,f**) Migration (**e**) and invasion (**f**) capabilities of cells treated with anti-CCL20 or anti-GM-CSF antibody. Cell migration and invasion were measured by wound-healing and transwell invasion assays, respectively. The data are expressed as the mean ± s.e.m. *P < 0.05, **P < 0.01 *versus* cells without anti-CCL20 or anti-GM-CSF.

Breast cancer-mediated bone loss is triggered by factors secreted from bone metastatic cells, and these factors accelerate osteolysis by promoting crosstalk among cancer cells, osteoblasts, and osteoclasts, creating a vicious cycle^2^. Chemokines greatly contribute to these cell-cell interactions^27^. We attempted to identify chemokines that are regulated by HuR and participate in this cycle using a Human Cytokine Array C6, which is composed of a nitrocellulose membrane spotted with antibodies specific to 60 different cytokines. The levels of CCL20 and granulocyte-macrophage colony-stimulating factor (GM-CSF) were decreased by 57.2% and 65.1%, respectively, in conditioned medium from shHuR MDA-MB-231 cells compared with that from shNC cells, whereas the levels of other cytokines did not indicate substantial inhibition (Fig. 2a and Supplementary Fig. 2a). In addition, the production of CCL20 and GM-CSF in various breast cancer cell lines, including the luminal B and basal-like/triple-negative breast cancer subtypes, was also regulated by HuR. Relatively high CCL20 levels were detected in conditioned media from ZR-75-1, MDA-MB-231, and BT549 cells, and HuR knockdown inhibited these levels by 38.2%, 40.1%, and 38.5%, respectively. In addition, high GM-CSF levels were detected that were inhibited by 20.3% and 24.3% by HuR knockdown in MDA-MB-231 and BT549 cells, respectively (Fig. 2b). Furthermore, reduced expression of CCL20 and GM-CSF was observed in tumor tissues from mice intracardially injected with shHuR cells based on immunohistochemical examination (Fig. 2c). However, CCR6 expression did not change by HuR knockdown (Supplementary Fig. 2b).

To clarify whether CCL20 and GM-CSF are critical factors that mediate the function of HuR in breast cancer bone metastasis, we added antibodies specific to these chemokines to culture media of MDA-MB-231 cells. Treatment with up to 500 ng/ml of anti-CCL20 and anti-GM-CSF antibody for 24 h slightly reduced cell viability by 19.3% and 23.4%, respectively (Fig. 2d). Additionally, treatment with these antibodies inhibited cell migration by 14.2% and 45.1% at 5 ng/ml and by 64.8% and 54.7% at 50 ng/ml (Fig. 2e). Cell invasion was also inhibited by 60.1% and 14.3% in response to neutralization with the anti-CCL20 and anti-GM-CSF antibodies at 5 ng/ml, respectively, and by close to 100% in response to antibody neutralization at 50 ng/ml (Fig. 2f). Furthermore, treatment with the CCL20 and GM-CSF chemokines rescued the metastatic ability of HuR-knockdown cells. Although the treatments with these chemokines did not markedly affect cell viability (Supplementary Fig. 3a), they did distinctly induce the migration (Supplementary Fig. 3b) and invasion (Supplementary Fig. 3c) of shHuR MDA-MB-231 cells in a dose-dependent manner.

HuR has been reported to directly bind to the AREs in the 3′-UTR of GM-CSF mRNA, particularly to the class II AREs, which are characterized by multiple overlapping AUUUA motifs^18, 37^. However, it is unknown whether HuR has a role in stabilizing CCL20 mRNA. We found that the human CCL20 mRNA contains three scattered AUUUA motifs, similar to the class I AREs present in early-response gene-encoding mRNAs (Supplementary Fig. 4a). RNA-based electrophoretic mobility shift assay (REMSA) was performed using biotin-labeled RNA probes containing a HuR-binding motif in the 3′-UTR of CCL20 mRNA. The results showed that the labeled RNA probes formed a complex with HuR in lysates from the shNC MDA-MB-231 cells and that the formation of this complex was significantly decreased in lysates from the shHuR cells (Supplementary Fig. 4b). The binding of HuR to RNA probes was blocked in the presence of an excess amount of unlabeled oligonucleotides. These data demonstrate that HuR regulates CCL20 production by directly binding to the 3′-UTR of CCL20 mRNA. Taken together, our results suggest that HuR in breast cancer cells regulates the production of CCL20 and GM-CSF and that the secretion of these chemokines promotes cancer cell migration and invasion.

### Gene expression of CCL20 rather than that of GM-CSF is highly correlated with gene expression of HuR, distant metastasis-free survival, and overall survival in breast cancer

**Figure 3:**
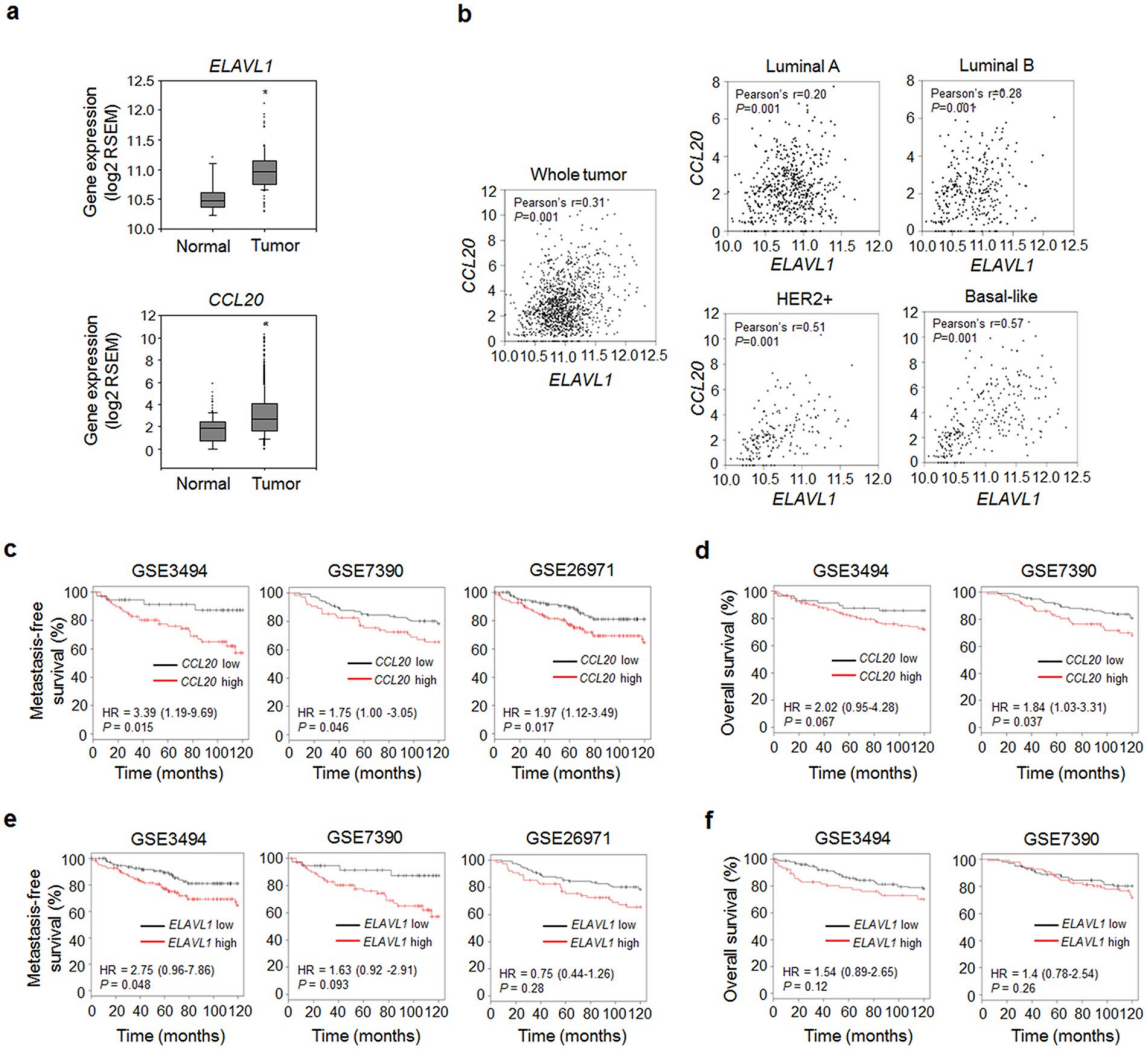
*CCL20* expression is correlated with *ELAVL1* expression, distant metastasis-free survival, and overall survival in patients with breast cancer. (**a**) Expression levels of *ELAVL1* and *CCL20* in normal and tumor tissues. The data were obtained from the TCGA database. RSEM: RNA-Seq by Expectation Maximization. *P < 0.01 *versus* normal breast tissues. (**b**) Scatterplot showing the correlations between *ELAVL1* and *CCL20* expression in whole breast cancer tissues and in tissues with different tumor subtypes. Pearson’s correlation analysis was performed to assess statistical significance. Normal: normal breast tissues (*n* = 113); Tumor: whole tumor tissues (*n* = 1,069); Luminal A subtype (*n* = 422); Luminal B subtype (*n* = 194); HER2-enriched subtype (*n* = 68); Basal-like subtype (*n* = 142). (**c–f**) Kaplan-Meier plots derived from clinical datasets (GSE3494: *n* = 130; GSE7390: *n* = 198; GSE26971: *n* = 256) showing the distant metastasis-free survival (**c,e**) and overall survival (**d,f**) of breast cancer patients with high or low *CCL20* expression (**c,d**) and with high or low *ELAVL1* expression (**e,f**). *P* values were determined using the log-rank test. Hazard ratios (HRs) with 95% confidence intervals are shown.

To evaluate the clinical relevance of our results, we analyzed breast cancer cases from the Cancer Genome Atlas (TCGA) database. The *ELAVL1* (the gene that encodes HuR) and *CCL20* expression levels were both markedly increased in breast tumor tissues compared with normal breast tissues (Fig. 3a). In contrast, neither *CSF2* (the gene that encodes GM-CSF) nor *CCR6* (the gene that encodes the only known receptor for CCL20) expression was upregulated (Supplementary Fig. 5a). In addition, *ELAVL1* expression showed a strong positive correlation with *CCL20* expression (Fig. 3b) but not with *CSF2* (Supplementary Fig. 5b) or *CCR6* expression (Supplementary Fig. 5c) in breast cancer subtypes, particularly the basal-like subtype. Kaplan-Meier survival analysis of GSE3494, GSE7390 and GSE26971 showed that metastasis-free survival (Fig. 3c) and overall survival (Fig. 3d) were decreased in the breast cancer patients with high *CCL20* expression. In contrast, no correlation was detected between high *ELAVL1*, *CSF2*, or *CCR6* expression and metastasis-free survival (Fig. 3e, Supplementary Fig. 5d,f) or overall survival (Fig. 3f, Supplementary Fig. 5e,g). These results suggest that breast cancer patients with upregulated CCL20 expression are at a high risk of metastasis.

### Anti-CCL20 antibody treatment prevents bone metastasis of breast cancer cells in mice

**Figure 4:**
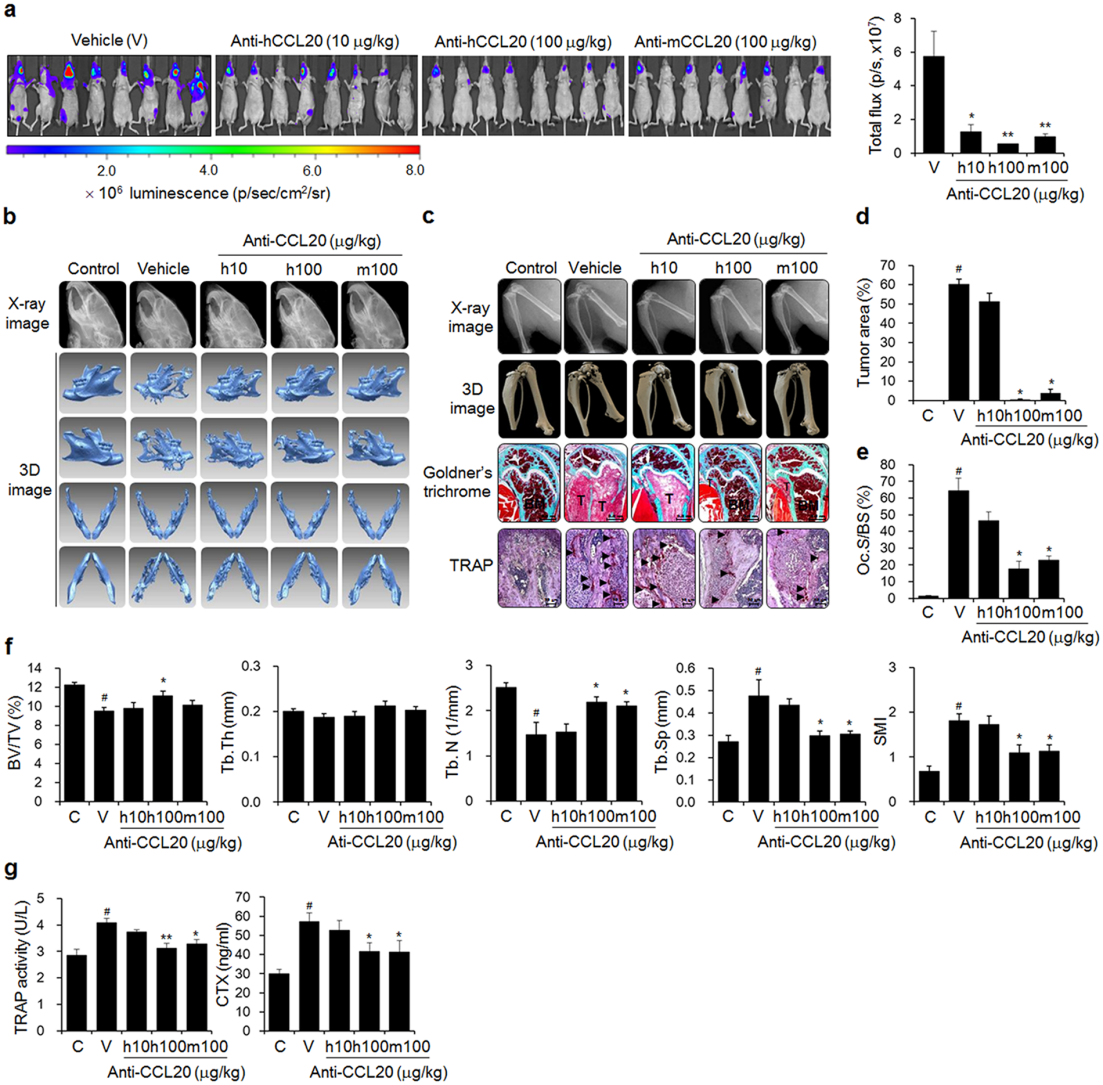
Anti-CCL20 treatment blocks bone metastasis of breast cancer cells in mice. (**a**) Metastatic progression detected by bioluminescence. Mice (*n* = 10) were injected with luciferase-transfected MDA-MB-231 cells into the left cardiac ventricles, followed by intraperitoneal administration of anti-human or anti-mouse CCL20 antibody at the indicated doses three times per week for 6 weeks. The control mice were treated with HBSS and PBS instead of the cancer cell suspension and anti-CCL20 antibody, respectively. The vehicle group was administered PBS instead of anti-CCL20. (**b,c**) X-ray and 3D images of mandibles (**b**), femora, and tibiae **(c)** derived from μCT scans. **(c)** Goldner’s trichrome and TRAP staining of femoral tissue sections. Arrowheads: TRAP-positive osteoclasts; T: tumor; B: bone; BM: bone marrow. Scale bar: 0.5 mm for Goldner’s trichrome staining and 10 μm for TRAP staining. (**d,e**) Tumor areas and (**d**) Oc.S/BS (**e**) values for stained femoral sections. (**f**) Bone morphometric parameters of the femora, including the BV/TV (%), Tb.Th (mm), Tb.N (1/mm), Tb.Sp (mm), and SMI. (**g**) Serum levels of the bone resorption markers TRAP 5b and CTX. The data are expressed as the mean ± s.e.m. ^#^ *P* < 0.05 *versus* control mice (C); *P < 0.05, **P < 0.01 *versus* vehicle-treated mice (V).

Although NF-kB-regulated GM-CSF has been implicated in osteolytic bone metastasis in mice injected with MDA-MB-231 breast cancer cells^38^, the role of CCL20 has not yet been determined. To assess whether CCL20 could serve as a novel therapeutic target for bone metastasis of breast cancer cells, we inoculated luciferase-transfected MDA-MB-231 cells into the left cardiac ventricles of nude mice. Then, we intraperitoneally administered either anti-human CCL20 antibody to target inoculated human cancer cell-derived CCL20 or anti-mouse CCL20 antibody to target surrounding non-cancer cell-derived CCL20 to the mice for 6 weeks. MDA-MB-231 cell metastasis was significantly inhibited by treatment with each antibody (Fig. 4a). In particular, treatment with each antibody at 100 μg/kg notably decreased the production of osteolytic lesions in the mandibles (Fig. 4b), distal femora, and proximal tibiae (Fig. 4c) of the MDA-MB-231 cell-injected mice, as shown by X-ray and 3D images derived from μCT scans. In addition, these treatments resulted in considerable decreases in tumor volume and bone destruction, as demonstrated by Goldner’s trichrome staining (Fig. 4c,d), and the reduced production of TRAP-positive activated osteoclasts on bone surfaces close to cancer cells (Fig. 4c,e). Intraperitoneal administration of anti-CCL20 antibody at 100 μg/kg inhibited the decreases in the BV/TV, Tb.Th, and Tb.N and the increases in Tb.Sp and SMI compared to those of the vehicle treated-mice (Fig. 4f). The antibody treatments also suppressed the elevated serum TRAP 5b and CTX levels caused by MDA-MB-231 cell inoculation (Fig. 4g). These results suggest that CCL20 is a pivotal factor for osteolytic breast cancer bone metastasis.

### CCL20 promotes the metastatic ability of breast cancer cells

**Figure 5:**
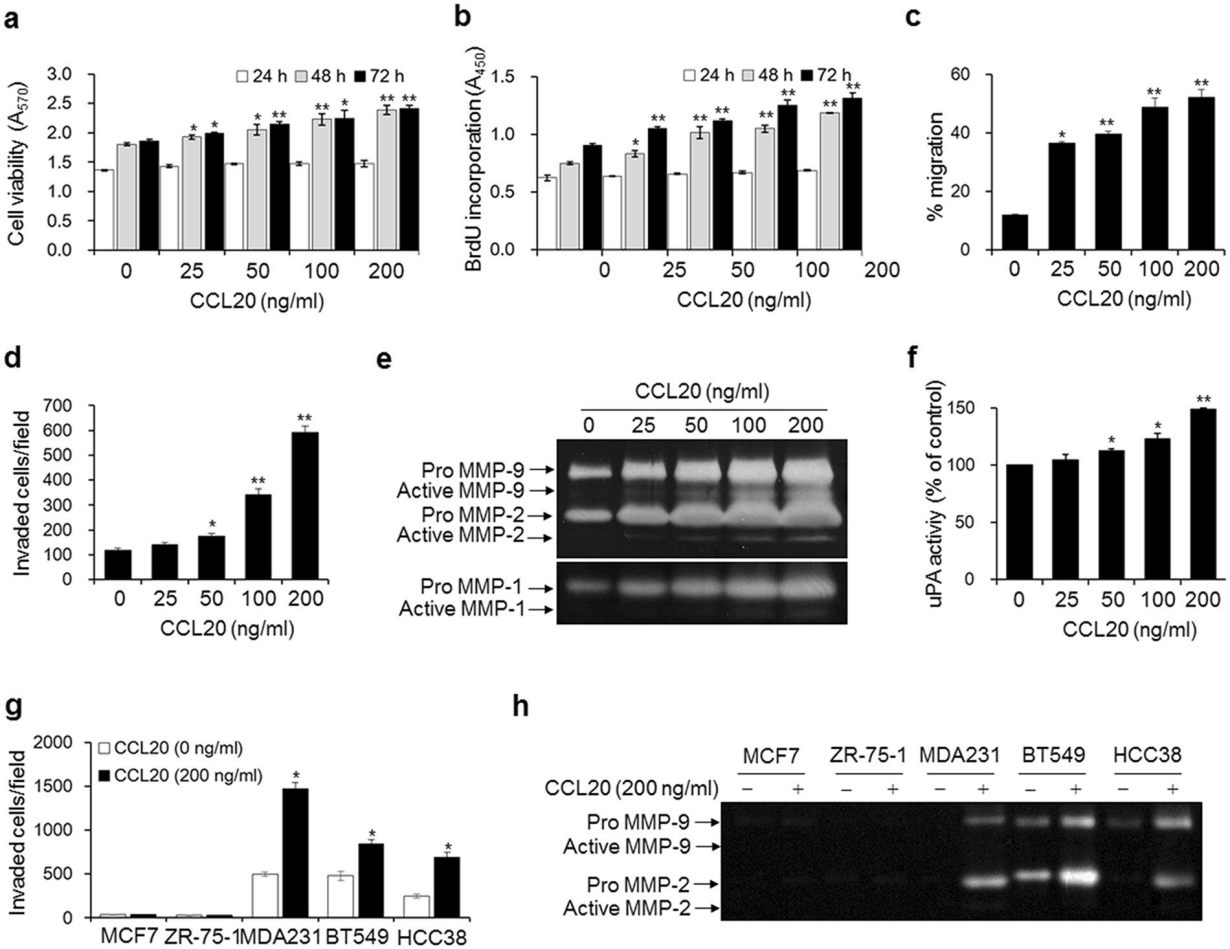
CCL20 enhances the metastatic ability of breast cancer cells. (**a**) Viabilities of breast cancer cells stimulated with the indicated concentrations of CCL20 for 24, 48, and 72 h. (**b–d**) Quantity of 5-bromo-2′-deoxyuridine (BrdU) incorporated into newly synthesized DNA (**b**), migration (**c**), and invasion (**d**) in MDA-MB-231 cells stimulated with the indicated concentrations of CCL20 for 24 h. (**e,f**) MMP-1, MMP-2/9 (**e**), and uPA (**f**) activities in conditioned media from MDA-MB-231 cells stimulated with the indicated concentrations of CCL20 for 24 h. MMP-1 and MMP2/9 activities were determined by collagen and gelatin zymography, respectively. The clear bands indicate the activities of the MMPs. uPA activity was measured with a commercial uPA activity assay kit. (**g,h**) Invasion (**g**) and MMP-2 and MMP-9 activities in conditioned media (**h**) of MCF-7, ZR-75-1, BT549, and HCC38 breast cancer cells stimulated with CCL20 (200 ng/ml) for 24 h. The data are expressed as the mean ± s.e.m. *P < 0.05, **P < 0.01 *versus* cells without CCL20.

To examine how CCL20 contributes to bone metastasis of breast cancer cells, we evaluated the effects of CCL20 treatment on the proliferation (Fig. 5a,b), migration (Fig. 5c), and invasion (Fig. 5d) of MDA-MB-231 cells. These processes were all dose-dependently increased in response to the treatment. In particular, cell migration and invasion were markedly increased. Degradation of the extracellular matrix by proteinases, such as MMPs and uPA, is pivotal for the invasion and metastasis of cancer cells. We found that MMP-1 and MMP-2/9 activities were markedly enhanced in conditioned medium from CCL20-treated MDA-MB-231 cells, as demonstrated by collagen and gelatin zymography, respectively (Fig. 5e). uPA activity was also increased by 38.3% in MDA-MB-231 cells treated with 200 ng/ml CCL20 (Fig. 5f). In addition, treatment with 200 ng/ml CCL20 significantly enhanced cell invasion (Fig. 5g) and stimulated the secretion of MMP-2 and MMP-9 (Fig. 5h) in BT549 and HCC38 cell lines, which are basal-like/triple-negative breast cancer cell lines, as is the MDA-MB-231 cell line. In contrast, the invasiveness of CCL-20-treated MCF-7 and ZR-75-1 luminal breast cancer cells was not enhanced, and no MMP activity was detected. These results demonstrate that CCL20 has greater effects on aggressive and highly metastatic breast cancer cell lines than on less metastatic breast cancer cell lines.

### CCL20 induces RANKL expression and mediates interactions between breast cancer cells and osteoblastic cells

**Figure 6:**
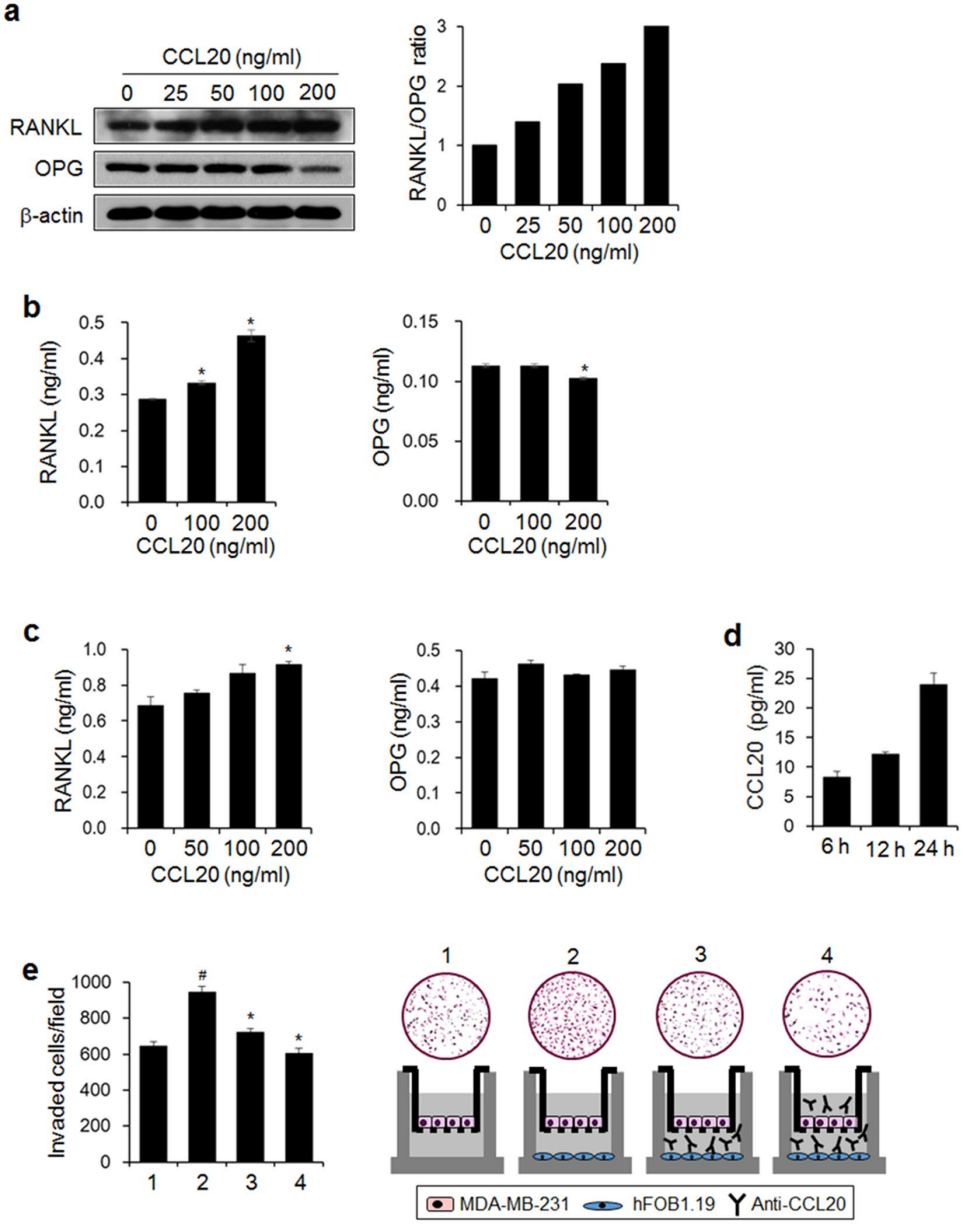
CCL20 increases the RANKL/OPG ratio and mediates interactions between human breast cancer cells and osteoblastic cells. **(a)** RANKL and OPG protein expression in MDA-MB-231 cells stimulated with the indicated concentrations of CCL20 for 24 h. Protein expression was detected by western blotting. β-actin served as a loading control. The graph presents the ratio of the intensity of RANKL to that of OPG after normalization against the intensity of β-actin. **(b)** Levels of RANKL and OPG in conditioned media from CCL20-stimulated MDA-MB-231 cells. **(c)** Levels of RANKL and OPG in conditioned media from CCL20-stimulated hFOB1.19 osteoblastic cells. (**d**) Levels of osteoblastic-derived CCL20 at the indicated time points. **(b–d)** RANKL, OPG, and CCL20 levels in conditioned media were detected with commercially available ELISA kits. The data are expressed as the mean ± s.e.m. *P < 0.05 *versus* cells without CCL20. **(e)** The effect of anti-CCL20 antibody on the invasion of MDA-MB-231 cells exposed to conditioned medium of osteoblastic cells. 1: MDA-MB-231 cells (upper)-media (lower); 2: MDA-MB-231 cells (upper)-hFOB1.19 cells (lower); 3: MDA-MB-231 cells (upper)-hFOB1.19 cells and anti-CCL20 antibody (lower); 4: MDA-MB-231 cells and anti-CCL20 antibody (upper)-hFOB1.19 cells and anti-CCL20 antibody (lower). ^#^ *P* < 0.05 *versus* 1; *P < 0.01 *versus* 2.

RANKL regulates the differentiation and activity of osteoclasts through its receptor RANK and is thus a key factor for bone metastasis^2, 39^. Breast cancer cells can both produce RANKL directly^40^ and affect osteoblastic RANKL expression by secreting osteolytic factors^41^. This induction of osteoclastogenesis results in cancer-related bone loss. We found that CCL20 enhanced the RANKL/OPG ratio by stimulating RANKL expression and secretion and reducing OPG expression and secretion in MDA-MB-231 human breast cancer cells in a dose-dependent manner (Fig. 6a,b). CCL20 also increased the RANKL level in the culture medium of hFOB1.19 human osteoblastic cells but did not change the secreted OPG level (Fig. 6c). Further, the osteoblastic cells produced CCL20 (Fig. 6d), and the antibody-mediated neutralization of osteoblastic cell-derived CCL20 significantly inhibited the invasiveness of MDA-MB-231 breast cancer cells (Fig. 6e). These results suggest that CCL20 produced by breast cancer cells elevates the osteoblastic RANKL/OPG ratio and that osteoblastic CCL20 also contributes to the invasiveness of breast cancer cells.

## Discussion

Controlling both the metastatic potential of cancer cells and the vicious cycle of bone metastase-induced bone destruction may be the best strategy to improve the survival and quality of life of patients with bone-tropic cancer. HuR plays crucial roles in essential steps in the metastatic process^21^ and is closely associated with the poor prognoses of lung, ovarian, colorectal, and breast cancer patients^23, 25, 42^. The development and progression of cancer-related skeletal diseases depends on the production of soluble factors within the metastatic niche that cooperate to promote the bone-resorbing functions of osteoclasts and to trigger interactions between cancer cells and surrounding cells^43^. Chemokines are very likely to represent these factors.

We attempted to determine the role of HuR in breast cancer bone metastasis and to identify novel osteolytic factors with high potentials as therapeutic targets from HuR-regulated chemokines. We demonstrated that HuR expression promoted breast cancer bone metastasis by considerably reducing the tumor burden and osteolysis in murine models inoculated with HuR-knockdown breast cancer cells. These findings were supported by the reduced metastatic potential of HuR-knockdown MDA-MB-231 cells. We further identified that the metastatic ability of HuR-expressing breast cancer cells was related to their secretion of CCL20 and GM-CSF. However, in our analysis using public databases, expression of the GM-CSF-encoding gene *CSF2* was not strongly correlated with that of the HuR-encoding gene *ELAVL1* in the different molecular subtypes of breast cancer. Additionally, neither distant metastasis-free survival nor overall survival was significantly correlated with *CSF2* expression in the breast cancer patients. In contrast, *CCL20* expression was significantly correlated with the above analyses. Thus, CCL20 may be a better target for breast cancer bone metastasis than GM-CSF.

Recombinant G-CSF/GM-CSF, which has hematopoietic activity, is routinely used to reverse leukopenia due to chemotherapy and radiation and as an adjuvant to boost the immunogenicity of tumor cells for diminishing drug resistance in anti-tumor therapy^44^. In contrast, G-CSF/GM-CSF has also been reported to promote cancer cell dissemination and bone metastasis via increased osteoclast formation and the subsequent homing of malignant cells to bone tissues^38, 45^. The roles of CCL20 in cancer development, tumor promotion, and metastasis are linked to the attraction of CCR6-expessing regulatory T lymphocytes to tumor sites expressing CCL20^46–49^ and to the recruitment of CCR6-expressing cancer cells to metastatic sites with abundant CCL20^50^. CCL20 expression has been reported to be upregulated in osteoblasts and osteoclast precursors co-cultured with multiple myeloma cells, and this upregulation contributes to the formation of osteoclasts and osteolytic bone lesions in multiple myeloma patients^51^. Our data verified that suppression of the functions of CCL20 molecules derived from cancer cells and surrounding non-cancer cells inhibited tumor growth in bone marrow, as well as severe osteolysis, in mice inoculated with MDA-MB-231 cells through the left cardiac ventricles. In addition, CCL20 markedly increased cell migration and invasion and the activities of related proteases in basal-like/triple-negative breast cancer cell lines but not in luminal breast cancer cell lines, suggesting that the targeting of CCL20 may be more effective in patients with aggressive breast cancer.

RANKL and OPG, which are both expressed by osteoblasts/stromal cells, are critical regulators of osteoclast differentiation. The binding of RANKL to its receptor, RANK, on osteoclast precursor cells stimulates osteoclast differentiation; however, its decoy receptor, OPG, blocks osteoclast differentiation by binding to RANKL^52, 53^. An abnormally increased RANKL/OPG ratio can lead to excessive osteoclastogenesis and subsequent bone resorption. Recent studies have also reported a critical role of RANKL in the development and progression of breast cancer^40, 54^. We found that breast cancer cells and bone cells influence each other in the bone microenvironment by producing CCL20; the RANKL/OPG ratio was increased in both MDA-MB-231 cells and human osteoblast hFOB1.19 cells exposed to CCL20, and osteoblast-derived CCL20 promoted breast cancer cell invasiveness. Thus, CCL20 may be a key factor for the acceleration of breast cancer metastasis and related bone loss.

Collectively, CCL20, which is regulated by HuR and secreted into the bone microenvironment, enhances the metastatic ability and bone metastasis of breast cancer cells by stimulating cancer cells and osteoblastic cells in both autocrine and paracrine manners. Thus, CCL20 may have high potential as a therapeutic target in breast cancer patients with bone metastasis.

## Methods

More details of reagents, antibodies, cell lines, and cell culture conditions were described in Supplementary materials and methods.

### HuR knockdown in breast cancer cells

Luciferase-transfected MDA-MB-231 cells (lucMDA-MB-231) were established as previously described^55^. LucMDA-MB-231 cells (1 × 10^5^ cells/dish) were seeded in 60-mm culture dishes. LucMDA-MB-231 cells at approximately 50% confluence were infected with HuR lentiviral particles (Santa Cruz) for knockdown of HuR (shHuR) or with control lentiviral particles (Santa Cruz) as a negative control (shNC) in DMEM containing 10% FBS and 10 μg/ml polybrene for 24 h. The infected cells were then cultured in fresh DMEM containing 10% FBS for 24 h. To select stable HuR-knockdown clones, the lentiviral particle-infected MDA-MB-231 cells were maintained in DMEM containing 10% FBS and puromycin (10 µg/ml) for an additional 2 weeks. In addition, MCF-7, ZR-75-1, MDA-MB-231, BT549, and HCC38 cells (3 × 10^5^ cells/well) were transfected with either negative control siRNA (Santa Cruz) or HuR siRNA (Santa Cruz) using Lipofectamine RNAiMAX reagent according to the manufacturer’s instruction.

### *In vivo* models

Female 5-week-old BALB/c *nu*/*nu* mice (NARA Biotech, Seoul, Korea) were provided access to commercial rodent chow and tap water *ad libitum* and housed under specific pathogen-free conditions with a relative humidity of 50 ± 5% and a 12-h light/dark cycle at 22 ± 2 °C. All animal experimental procedures were conducted in compliance with the guidelines and regulations for the use and care of animals established by Yonsei University College of Dentistry (Approval No. 2015-0255). All methods were carried out in accordance with relevant guidelines and regulations.

The role of HuR in breast cancer bone metastasis was determined in mice intracardially or intratibially injected with breast cancer cells^55–57^. Ten BALB/c *nu*/*nu* mice were randomly assigned per group. The mice were anesthetized with a mixture of 30 mg/kg Zoletil (Virbac Laboratories, Carros, France) and 10 mg/kg Rompun (Bayer HealthCare Korea, Seoul, Korea) and were then placed in the supine position. A suspension of shNC or shHuR MDA-MB-231 cells (1 × 10^6^ cells/0.1 ml HBSS) was slowly inoculated into the left cardiac ventricle of each mouse using a 1-ml syringe with a 27-gauge needle. Alternatively, a suspension of shNC or shHuR MDA-MB-231 cells (1 × 10^6^ cells/50 μl HBSS) was injected into the tibia of each mouse using a 0.5-ml syringe with a 27-gauge needle. The control mice received PBS alone.

To investigate the role of CCL20 in breast cancer cell bone metastasis, exponentially growing lucMDA-MB-231 cells (1 × 10^6^ cells/0.1 ml HBSS) were injected into the left cardiac ventricles of mice (10 mice per group). The control mice received HBSS alone. The mice were intraperitoneally administered anti-human CCL20 (10 or 100 μg/kg) or anti-mouse CCL20 (100 μg/kg) antibody 3 times per week. The vehicle and control mice were injected with PBS alone.

After 5 (for the intratibially injected mice) or 6 weeks (for the intracardially injected mice), the mice were anesthetized for bioluminescence imaging^55^ and μCT analysis^55, 56^. Quantitative bioluminescence data were expressed as photons/sec/cm^2^/sr. Goldner’s trichrome and TRAP stainings and immunohistochemical analysis for HuR, CCL20, and GM-CSF were accomplished in the sections derived from mouse hind limbs as describe previously^55, 56^. Tumor areas and osteoclast surface per bone surface (Oc.S/BS) values were measured with IMT i-Solution software (version 7.3, IMT i-Solution, BC, Canada). Tumor areas were calculated as the percentage of total tumor area per tissue area. Oc.S/BS values were determined as the percentage of bone surface in contact with osteoclasts. The serum levels of TRAP 5b and CTX were measured with a mouse TRAP assay kit (Immuno Diagnostic Systems, Boldon, UK) and a RatLaps EIA kit (Immuno Diagnostic Systems) according to the manufacturer’s instructions.

### Cell viability, migration, and invasion

Cell viability, migration, and invasion were measured with MTT assay, wound-healing assay, and a transwell chamber (Corning Costar, Lowell, MA), respectively, as described in Supplementary materials and methods.

### Cytokine and chemokine array

shNC and shHuR MDA-MB-231 cells (5 × 10^5^ cells/dish) were seeded in 60-mm culture dishes containing 10% FBS-DMEM and incubated overnight. The cells were washed twice with PBS, cultured in serum-free DMEM for 24 h, and centrifuged at 200 × g for 5 min. Multiple cytokines in the collected conditioned media were detected using a RayBio Human Cytokine Ab Array 6 (Ray Biotech, Norcross, GA) according to the manufacturer’s instruction. The membranes used in the array were scanned, and the intensity of each cytokine was analyzed using TINA software (Raytest, Straubenhardt, Germany). The relative cytokine intensities were normalized using the positive control spots on the same membrane.

### REMSA

shNC and shHuR MDA-MB-231 cells (1 × 10^6^ cells/plate) were seeded into 100-mm culture dishes and cultured in DMEM supplemented with 10% FBS. Cell lysates were prepared using RIPA buffer containing 1 mM phenylmethylsulfonyl fluoride (PMSF, Millipore, Billerica, MA) and protease inhibitor cocktail. The cell lysates were centrifuged at 22,000 × g for 15 min at 4 °C, and the protein concentrations in the supernatants were determined using a BCA protein assay. The sequence of the probe for the HuR-specific binding was 5′-AUUUUAUGUUAUUUAUAGCUGUAGGUUU-3′ (563–590 nt). The probes were synthesized as single strands that were labeled at the 3′-end with biotin. Unlabeled oligonucleotides were used as competitors. REMSA was performed using a LightShift Chemiluminescent EMSA Kit (Pierce) according to the manufacturer’s protocol.

### Public database analysis

*ELAVL1*, *CCL20*, *CSF2*, and *CCR6* mRNA expression levels in normal and breast cancer tissues were retrieved from the TCGA Research Network website. Correlations of *ELAVL1* expression with *CCL20*, *CSF2*, and *CCR6* expression were reanalyzed using Statistical Package for the Social Sciences (SPSS) software (IBM, Endicott, NY). Additional details of the study are available on the TCGA Research Network website. Kaplan-Meier plots for the overall survival and metastasis-free survival of breast cancer patients were generated using the GSE3994, GSE7390, and GSE26971 datasets with Kaplan-Meier Plotter (http://kmplot.com/analysis/)^58^. The patient samples were split into a high expression group and a low expression group for each gene using the following setting of best cutoff. The JetSet best probe set was used to analyze *ELAVL1*, *CCL20*, *CSF2*, and *CCR6* expression.

### BrdU incorporation

MDA-MB-231 cells (1 × 10^4^ cells/well) were seeded into a 96-well plate and cultured in serum-free DMEM supplemented with CCL20 at the indicated concentrations for 24, 48, and 72 h. The cells were then treated with BrdU (10 µM) for 2 h. The amount of BrdU incorporated into the DNA of proliferating cells was quantified using a BrdU labeling and detection kit (Roche Diagnostics) according to the manufacturer’s protocol.

### Zymography and uPA activity

BT549, HCC38, MCF-7, MDA-MB-231, and ZR-75-1 cells (5 × 10^5^ cells/dish) were each seeded into 60-mm culture dishes and incubated with the indicated concentrations of CCL20 in serum-free medium for 24 h. The culture media were collected by centrifugation at 200 × g for 5 min. MMP activities in the collected media were determined on 8% polyacrylamide gels containing 0.8 mg/ml gelatin (for MMP-2 and MMP-9) or 1 mg/ml collagen (for MMP-1) as described previously^55^. The gels were stained with 0.1% Coomassie Blue R-250, and MMP activities were assessed by analyzing clear bands against a blue background. uPA activity was determined using a uPA activity assay kit (Chemicon, Billerica, MA) according to the manufacturer’s instruction.

### Western blot analysis

shNC and shHuR MDA-MB-231 cells (5 × 10^5^ cells/dish) were each cultured in 10% FBS-DMEM for 24 h. Total cell lysates were prepared using RIPA buffer containing 1 mM PMSF and protease inhibitor cocktail. The lysates were centrifuged and the protein concentrations in the supernatants were measured using a BCA kit. Equal amounts of each lysate (30 μg) were separated on sodium dodecyl sulfate-polyacrylamide gels. Target proteins were detected using a primary antibody (1:1000) against HuR, RANKL, OPG, or β-actin as described previously^55, 56^.

### Detection of secreted RANKL and OPG

MDA-MB-231 (1 × 10^4^ cells/well) and hFOB1.19 cells (1 × 10^5^ cells/well) were treated with CCL20 at the indicated concentrations in serum-free media for 24 h, and the media were collected. The RANKL and OPG levels in the culture media were determined using commercially available ELISA kits (EIAab, Guangguguoji, China) according to the manufacturer’s instructions.

### Statistical analysis

The data are expressed as the mean ± s.e.m. Statistical analyses were performed using one-way ANOVA and Student’s *t*-test to detect significant differences between two groups. A *P* < 0.05 was considered statistically significant. In public database analysis, the data retrieved from the TCGA website were reanalyzed using SPSS (IBM, Endicott, NY) to determine Pearson’s correlation coefficients (r) between *ELAVL1* expression and *CCL20*, *CSF2*, and *CCR6* expression. Hazard ratios (HRs) with 95% confidence intervals and *P* values were calculated using the log-rank test.

### Data availability

All data generated during this study are included in this published article and its Supplementary Information files. The datasets analyzed during the current study are available in the the TCGA Research Network website (http://cancergenome.nih.gov/).

## Electronic supplementary material

Supplementary information
