# Supplementary material for: Human antigen R-regulated CCL20 contributes to osteolytic breast cancer bone metastasis
Source: Sci Rep. 2017 Aug 29;7:9610. doi: 10.1038/s41598-017-09040-4 (PMC5575024; doi:10.1038/s41598-017-09040-4)

Human antigen R-regulated CCL20 contributes to osteolytic breast cancer bone metastasis

Sun Kyoung Lee[1,2], Kwang-Kyun Park[1,2], Hyun-Jeong Kim[1], Junhee Park[3], Seung Hwa Son[4], Ki Rim Kim[5] & Won-Yoon Chung[1,2,*]

[1]Department of Oral Biology, Oral Cancer Research Institute, BK21 PLUS Project, Yonsei University College of Dentistry, Seoul 03722, Republic of Korea.

[2]Department of Applied Life Science, The Graduate School, Yonsei University, Seoul 03722, Republic of Korea.

[3]Department of Dentistry, The Graduate School, Yonsei University, Seoul 03722, Republic of Korea.

[4]Department of Dental Hygiene, Gangdong College, Icheon, 27600, Republic of Korea

[5]Department of Dental Hygiene, College of Science and Engineering, Kyungpook National University, Sangju, 37224, Republic of Korea

Supplementary Materials and Methods

Reagents and antibodies. Dulbecco's modified Eagle's medium **(**DMEM), RPMI 1640, phosphate-buffered saline **(**PBS), heat-inactivated fetal bovine serum **(**FBS), Hanks' balanced salt solution (HBSS), Geneticin (G418), and 0.25% trypsin-EDTA were purchased from Gibco BRL (Grand Island, NY). Recombinant human CCL20, monoclonal anti-human and anti-mouse CCL20 antibodies, and mouse IgG1 were obtained from R&D Systems (Minneapolis, MN). Monoclonal anti-human GM-CSF and OPG antibodies were obtained from Abcam (Cambridge, UK). A polyclonal anti-RANKL antibody and RIPA buffer were purchased from Cell Signaling Technology (Danvers, MA), and a protease inhibitor cocktail tablet was purchased from Roche Diagnostics (Mannheim, Germany). A bicinchoninic acid (BCA) protein assay reagent kit was obtained from Pierce (Rockford, IL). A monoclonal anti-β-actin antibody, polybrene, puromycin, horseradish peroxidase (HRP)-conjugated secondary antibodies, and ECL reagents were obtained from Santa Cruz Biotechnology (Santa Cruz, CA). Mitomycin C, 3-(4,5-dimethylthiazol-2-yl)-2,5-diphenyltetrazolium bromide (MTT), and insulin were purchased from Sigma-Aldrich (St. Louis, MO). Matrigel was purchased from BD Biosciences (Palo Alto, CA). Blasticidin S, HRP-goat anti-rabbit IgG (H+L), and Lipofectamine RNAiMAX reagent were obtained from Invitrogen (Carlsbad, CA). All reagents used were of analytical grade.

Cell lines and culture. Two human luminal breast cancer cell lines (MCF-7 and ZR-75-1), three human basal-like triple-negative breast cancer cell lines (MDA-MB-231, BT549, and HCC38), and hFOB1.19 human fetal osteoblastic cells immortalized with SV40 large T antigen were purchased from the American Type Culture Collection (Manassas, VA). The MCF-7 and MDA-MB-231 cells were cultured in DMEM supplemented with 10% FBS, and the HCC38 and ZR-75-1 cells were grown in RPMI 1640 supplemented with 10% FBS. The BT549 cells were cultured in RPMI 1640 supplemented with 0.023 IU/ml insulin and 10% FBS. All breast cancer cell lines were maintained at 37°C in a humidified atmosphere of 5% CO_2. hFOB1.19 human fetal osteoblastic cells were cultured in phenol red-free DMEM/F12 with 10% FBS and 0.3 mg/ml G418 at 34°C in a humidified atmosphere of 5% CO_2.

Western blot analysis. HuR-expressing and knockdown breast cancer cells (5×10^5 cells/dish) were each cultured in 10% FBS-DMEM for 24 h. Total cell lysates were prepared using RIPA buffer containing 1 mM PMSF and protease inhibitor cocktail. The lysates were centrifuged and the protein concentrations in the supernatants were measured using a BCA kit. Equal amounts of each lysate (30 μg) were separated on sodium dodecyl sulfate-polyacrylamide gels. Target proteins were detected using a primary antibody (1:1000) against CCR6 or β-actin.

Cell migration. Cells (2×10^5 cells/well) were seeded into a 6-well plate and incubated in DMEM supplemented with 10% FBS. After reaching approximately 90% confluence, the cells were scratched once with a micropipette tip. To assess the effect of HuR knockdown, shNC and shHuR MDA-MB-231 cells were each incubated in DMEM containing 1% FBS and mitomycin C (0.5 μg/ml). To examine the effects of CCL20 and GM-CSF, MDA-MB-231 cells were treated with various concentrations of anti-human CCL20 or anti-human GM-CSF antibody, and shNC and shHuR cells were treated with the indicated concentrations of CCL20 or GM-CSF. After 24 h of treatment, wound healing was assessed by comparing photographs taken at the time of scratching and 24 h later using an Olympus IX70 inverted optical microscope (Olympus Optical, Tokyo, Japan). Areas of cell migration were analyzed using ImageJ software by measuring wound surface areas at 0 h and 24 h. The data were expressed as the percentage of wound closure using the following formula: (1–(current wound width/initial wound width)) × 100.

Cell invasion. The chamber was fitted with a 6.5-mm polycarbonate membrane with an 8.0-μm pore size, which was coated with gelatin (1 mg/ml in distilled water) on the lower surface and Matrigel (1 mg/ml in DMEM) on the upper surface. To determine the effect of HuR knockdown, shNC and shHuR MDA-MB-231 cells (2×10^4 cells/0.1 ml) were added to the Matrigel-coated upper chambers, and the lower chambers were filled with 0.6 ml DMEM containing 1% FBS. To assess the effects of CCL20 and GM-CSF on invasiveness, a suspension of MDA-MB-231 cells (2×10^4 cells/0.1 ml) mixed with anti-human CCL20 or GM-CSF antibody was added to the upper chambers, and the lower

chambers were filled with 0.6 ml DMEM containing 1% FBS and anti-human CCL20 or GM-CSF antibody. In addition, shNC and shHuR MDA-MB-231, BT549, HCC38, MCF-7, MDA-MB-231, or ZR-75-1 cells were added to the upper chambers, and the lower chambers were filled with 0.6 ml DMEM or RPMI 1640 containing 1% FBS and different concentrations of CCL20 or GM-CSF.

To determine the effect of osteoblast-secreted CCL20 on breast cancer cell invasiveness, MDA-MB-231 cells (2×10^4 cells/0.1 ml) with or without anti-human CCL20 (50 ng/ml) were added into the upper chambers. The lower chambers were filled with 0.6 ml 1% FBS-DMEM/F12 containing hFOB1.19 cells (1×10^5 cells/well) and/or anti-human CCL20 antibody (50 ng/ml). After 24 h of incubation, the invaded cells on the lower surfaces of the membranes were fixed with 70% methanol and stained with hematoxylin. The non-invaded cells on the upper surfaces of the membranes were gently wiped off using cotton swabs, and the membranes that contained invaded cells were mounted onto slides. The number of invaded cells was counted using a Zeiss Axio Imager Microscope (Carzeiss, Gottingen, Germany).

Supplementary figures and legends

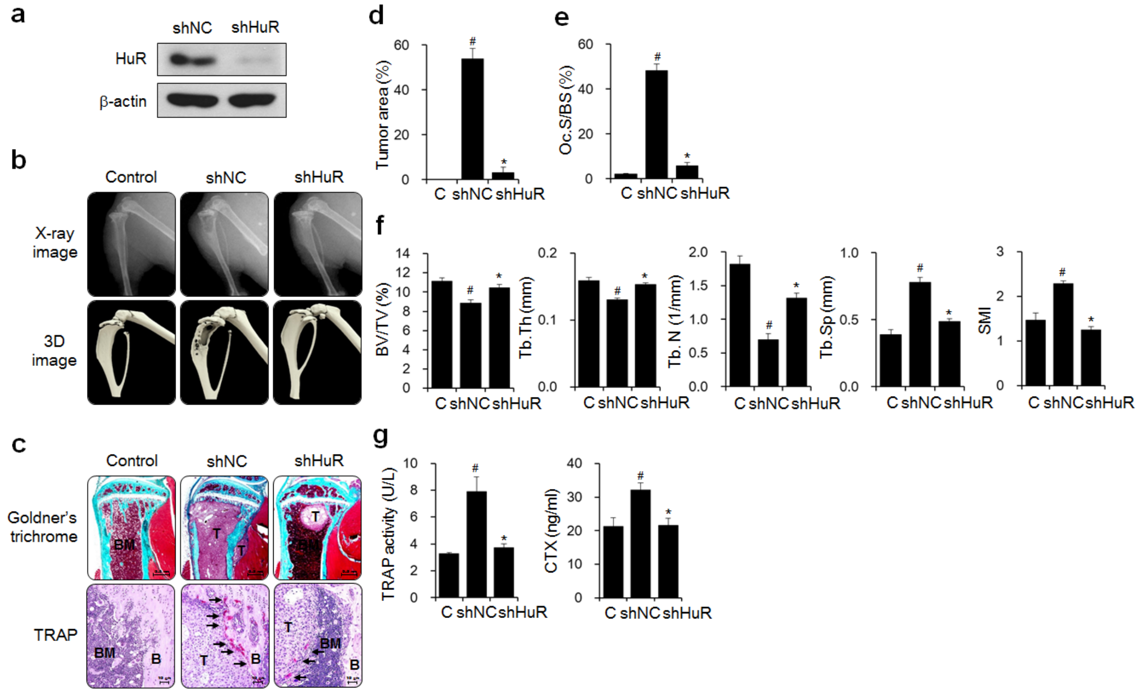

Figure S1. Effect of HuR knockdown on breast cancer-induced bone destruction in a murine intratibial model of bone metastasis. (a) HuR protein expression in shHuR and shNC MDA-MB-231 cells transduced with lentiviral particles containing either HuR shRNA or non-specific shRNA. Protein expression was determined by western blot analysis and β-actin was used as an internal control for protein loading. (b) Representative X-ray and 3D images of femora and tibiae from mice (*n* = 10/group) intratibially injected with shNC or shHuR MDA-MB-231 cells and then scanned using μCT after 5 weeks. (c) Goldner's trichrome and TRAP staining of femoral tissues. TRAP-positive osteoclasts are highlighted by arrowheads. T: tumor; B: bone; BM: bone marrow. Scale bar: 0.5 mm for Goldner's trichrome staining and 10 μm for TRAP staining. (d) Tumor areas and (e) Oc.S/BS values for stained femoral tissues. (f) Bone morphometric parameters of the femora at 5 weeks after inoculation of breast cancer cells. The BV/TV (%), Tb.Th (mm), Tb.N (1/mm), Tb.Sp (mm), and SMI in the mouse femora were analyzed by μCT. (g) Serum levels of TRAP 5b and CTX. The levels of the bone resorption markers were quantified using commercial kits at 5 weeks after the injection of breast cancer cells. The data are expressed as the mean ± s.e.m. $^{\#}P < 0.05$ *versus* control mice (C); $^{*}P < 0.01$ *versus* mice injected with shNC cells.

a

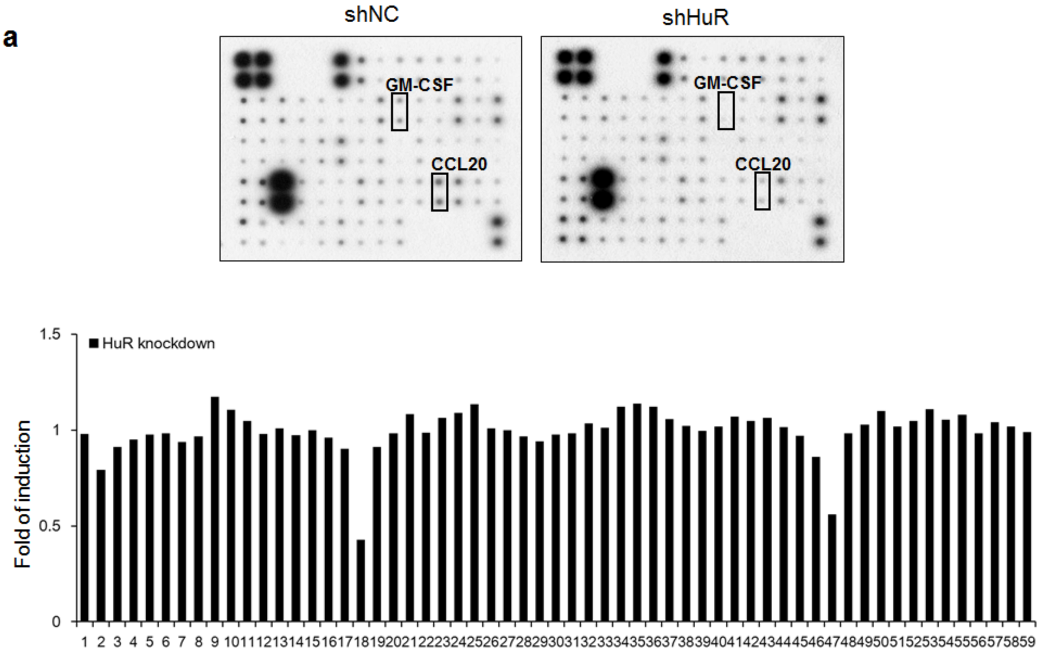

1	2	3	4	5	6	7	8	9	10
Angiogenin	BDNF	BLC	BMP-4	BMP-6	CK bg1	CNTF	EGF	Eotaxin-1	Eotaxin-2
11	12	13	14	15	16	17	**18**	19	20
Eotaxin-3	FGF-6	FGF-7	Fit-3 Ligand	Fractalkine	GCP-2	GDNF	**GM-CSF**	I-309	IFN-γ
21	22	23	24	25	26	27	28	29	30
IGFBP-1	IGFBP-2	IGFBP-4	IGF-I	IL-10	IL-13	IL-15	IL-16	IL-1α	IL-1β
31	32	33	34	35	36	37	38	39	40
IL-1ra	IL-3	IL-4	IL-5	IL-6	IL-7	Leptin	LIGHT	MCP-1	MCP-2
41	42	43	44	45	46	**47**	48	49	50
MCP-3	MCP-4	M-CSF	MDC	MIG	MIP-1δ	**MIP-3α**	NAP-2	NT-3	PARC
51	52	53	54	55	56	57	58	59	
PDGF-BB	RANTES	SCF	SDF-1	TARC	TGF-β1	TGF-β3	TNF-α	TNF-β	

b

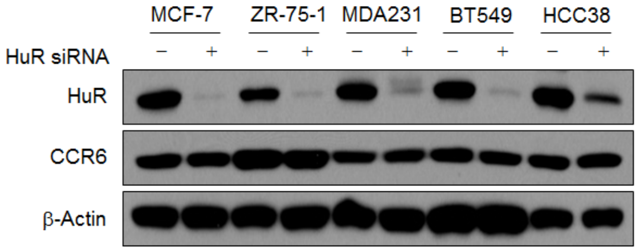

Figure S2. HuR-regulated cytokines and chemokines in MDA-MB-231 cells. (a) CCL20 and GM-CSF spots. Secretion patterns of 60 cytokines and chemokines in conditioned media (CM) of shNC or shHuR MDA-MB-231 cells were detected using a cytokine antibody array, as described in the Materials and Methods. The graphs show the fold changes in the expression of cytokines and chemokines in the CM of shHuR MDA-MB-231 cells compared to the CM of shNC MDA-MB-231 cells. (b) CCR6 expression in breast cancer cell lines. CCR6 protein levels were detected using Western blotting.

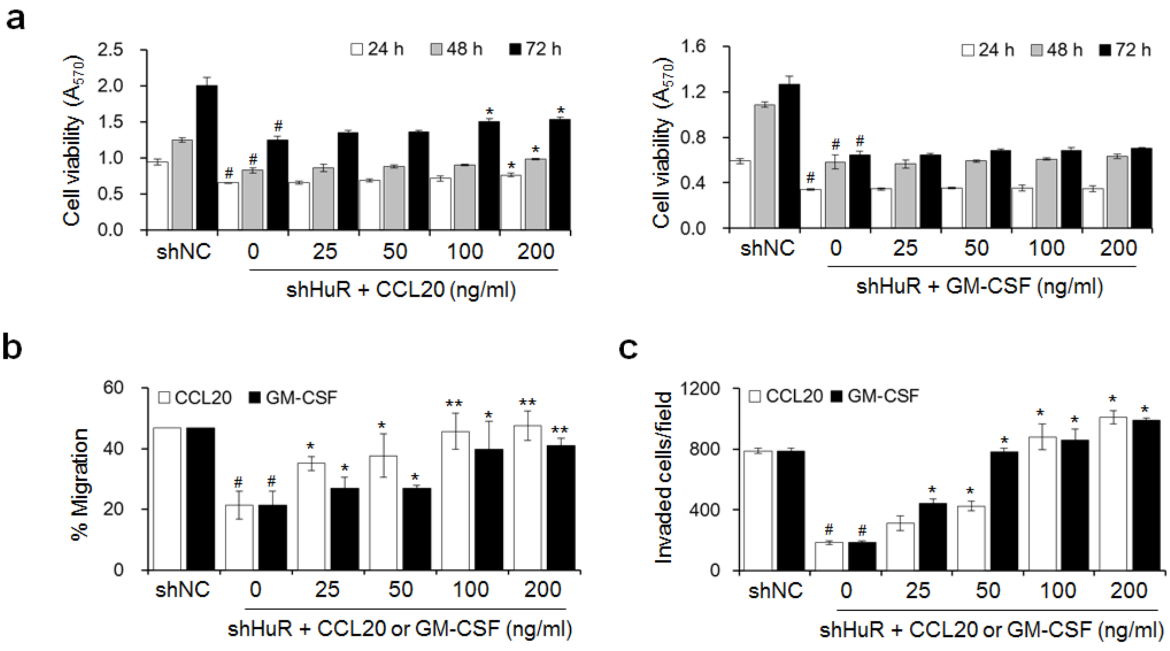

Figure S3. Effects of CCL20 and GM-CSF on the viability, migration, and invasiveness of shHuR MDA-MB-231 cells. (a) Viabilities of shHuR MDA-MB-231 cells treated with the indicated concentrations of CCL20 or GM-CSF for 24, 48, and 72 h. Cell viability was determined using MTT assay. (b) Migration and (c) invasiveness of shHuR MDA-MB-231 cells exposed to DMEM containing 1% FBS and the indicated concentrations of CCL20 or GM-CSF for 24 h. The data are expressed as the mean ± s.e.m. $^{\#}P < 0.01$ *versus* shNC cells; $^{*}P < 0.05$, $^{**}P < 0.01$ *versus* shHuR cells without CCL20 or GM-CSF.

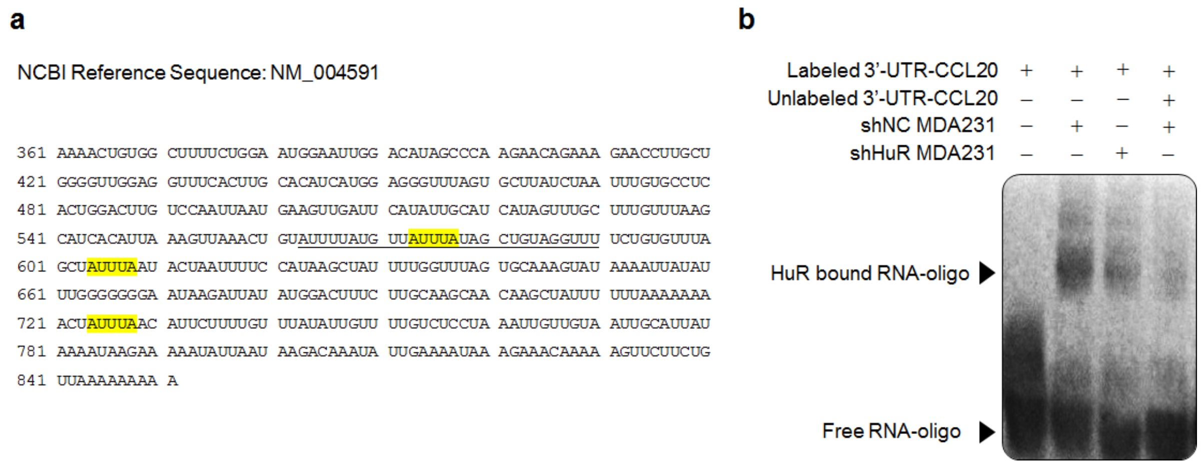

Figure S4. Binding ability of HuR to CCL20 mRNA. (a) CCL20 mRNA sequences (NCBI Reference Sequence: NM_004591). The areas surrounded by yellow boxes represent HuR binding sites, and the underlined part indicates the region of RNA that was prepared for REMSA. (b) The binding ability of HuR to the 3'-UTR of CCL20 mRNA was analyzed by REMSA as described in the Supplementary Materials and Methods.

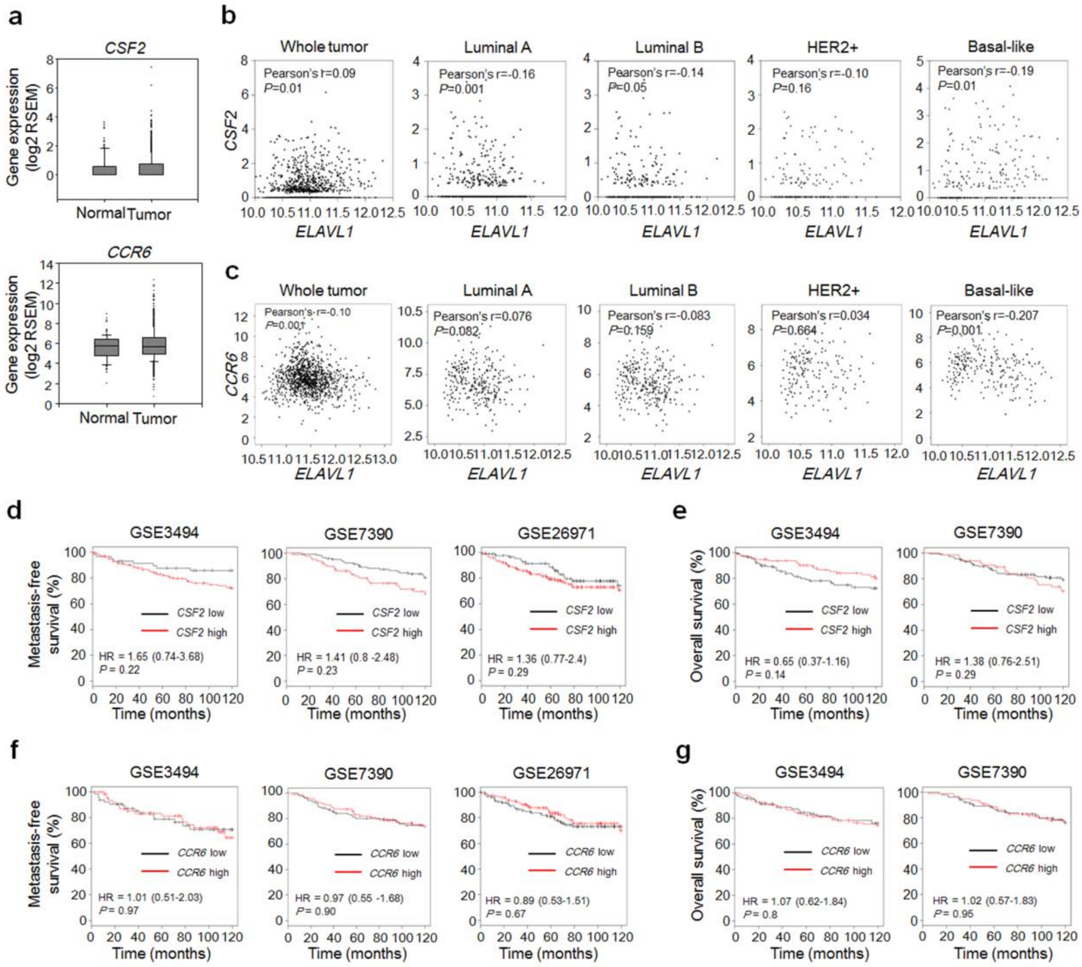

Figure S5. Clinical associations and correlations among *ELAVL1*, *CSF2*, and *CCR6* expression in breast cancer patients. (a) Expression levels of *CSF2* and *CCR6* in normal and tumor tissues. The data were obtained from the TCGA database. RSEM: RNA-Seq by Expectation Maximization. (b,c) Scatterplot showing the correlations of *ELAVL1* expression with *CSF2* (b) and *CCR6* (c) expression in whole breast cancer tissues and in tissues with different subtypes of breast cancer. Pearson's coefficient tests were performed to assess statistical significance. Normal breast tissues (n = 113), whole tumor tissues (n = 1,069), luminal A subtype (n = 422), luminal B subtype (n = 194), HER2-enriched subtype (n = 68), and basal-like subtype (n = 142). (d-g) Kaplan-Meier plots derived from clinical datasets (GSE3494: n = 130; GSE7390: n = 198; GSE26971: n = 256) showing the distant metastasis-free survival (d, f) and overall survival (e, g) of breast cancer patients with high or low *CSF2* expression (d, e) and with high or low *CCR6* expression (f, g). P values were determined using the log-rank test. HRs with 95% confidence intervals are shown.

Main Figure 5

MMP-2 (72 kDa latent and 66/62 kDa active forms)

MMP-9 (92kDa human latent and 88 kDa active forms)

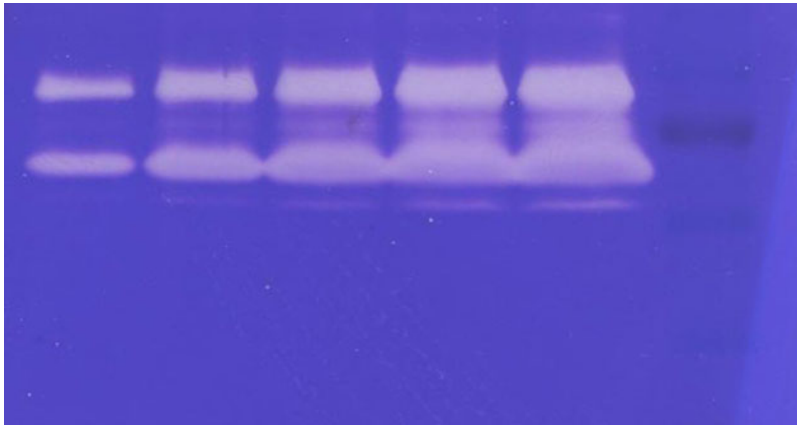

MMP1 (57/52 kDa latent and 49/37 kDa active forms)

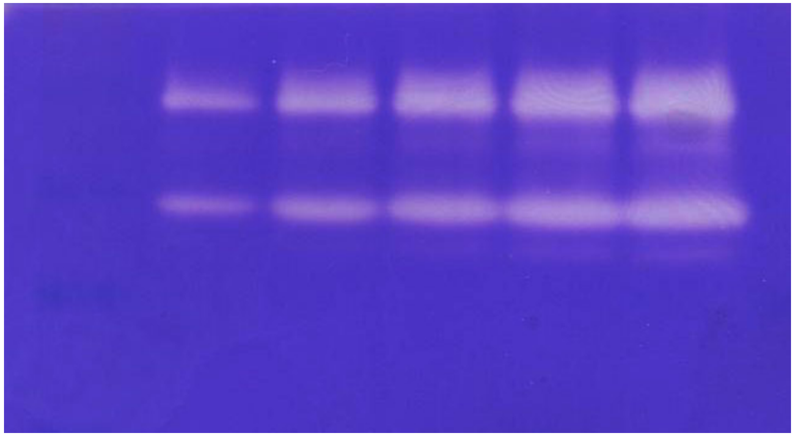

Main Figure 5

MMP-2 (72 kDa latent and 66/62 kDa active forms)

MMP-9 (92kDa human latent and 88 kDa active forms)

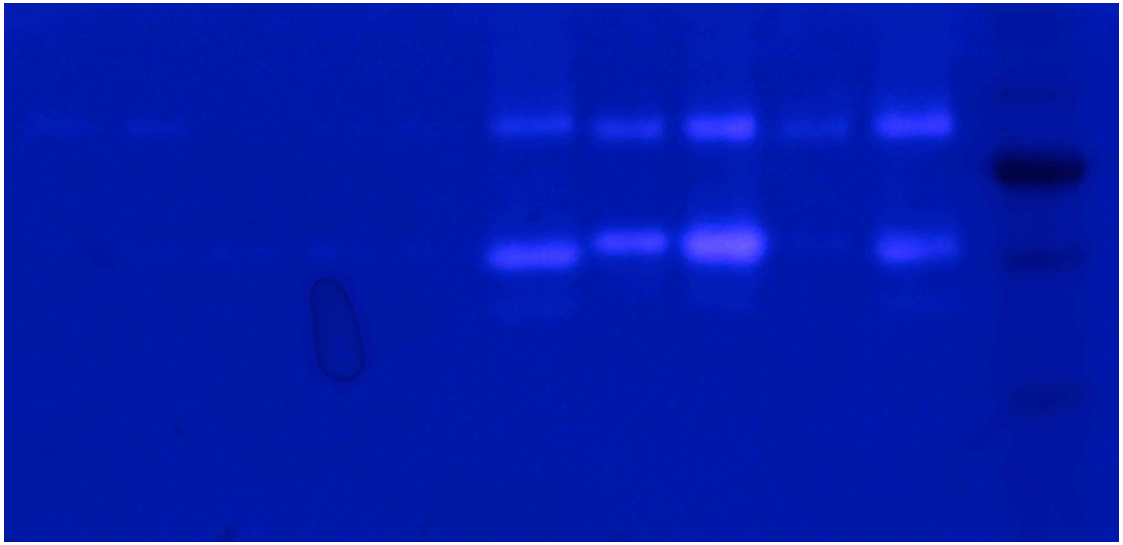

Main Figure 6

RANKL

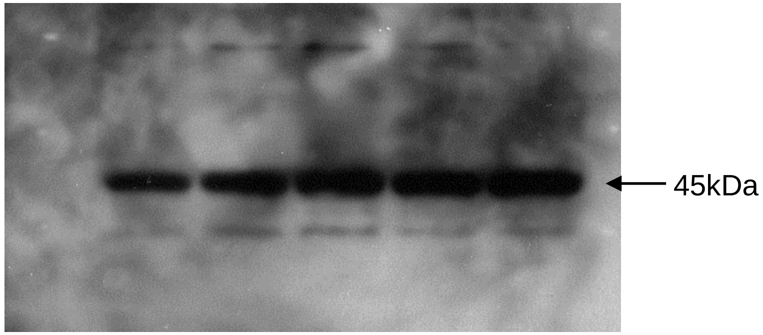

45kDa

OPG

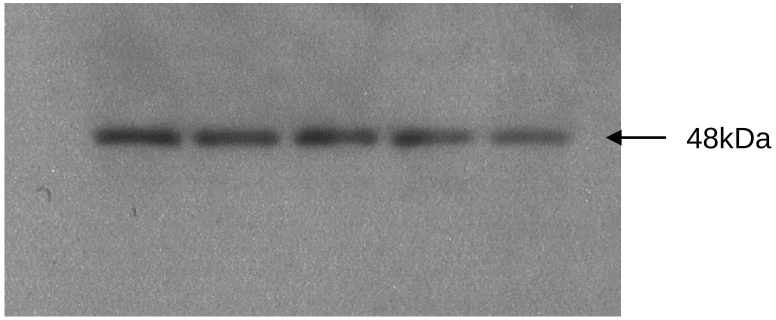

48kDa

β-Actin

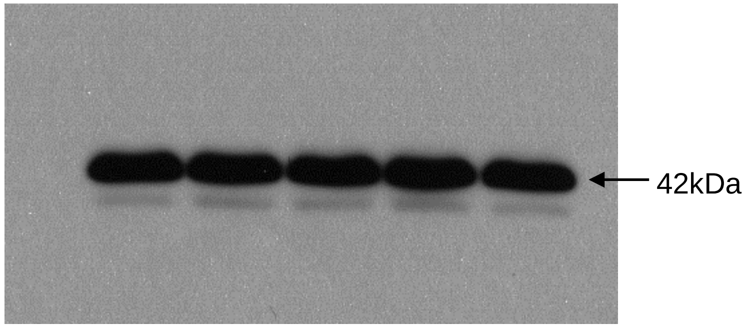

42kDa

Supplemental Figure 1

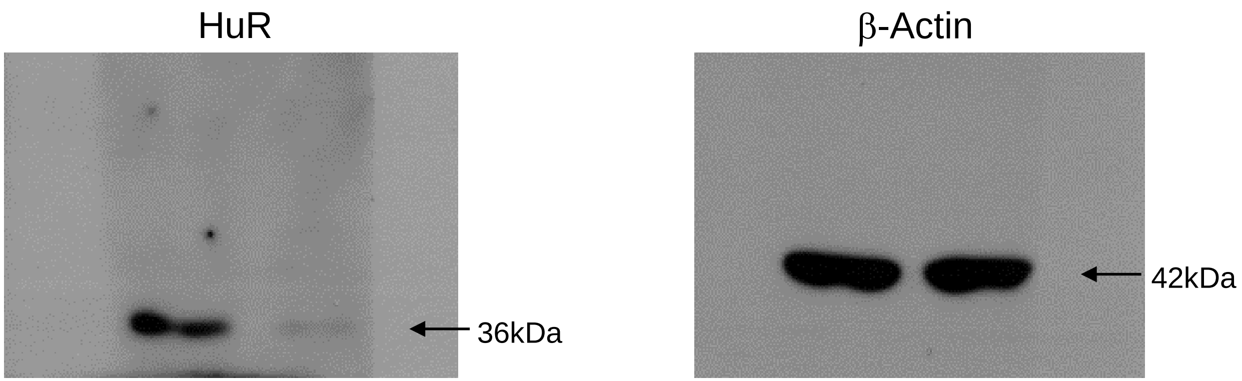

Supplemental Figure 2

HuR

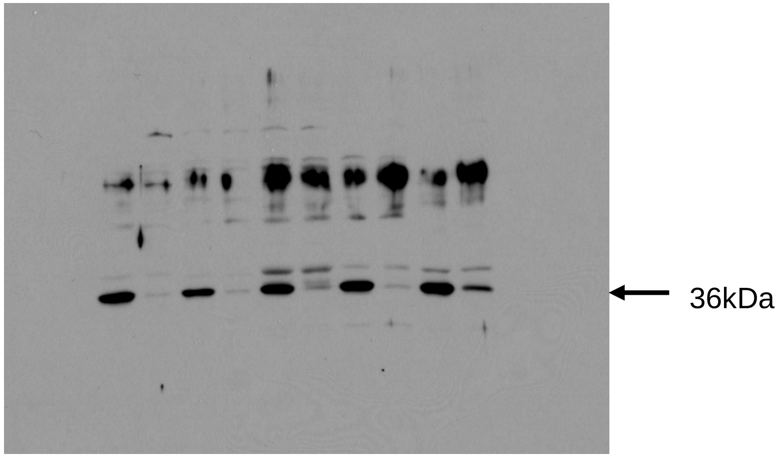

β-Actin

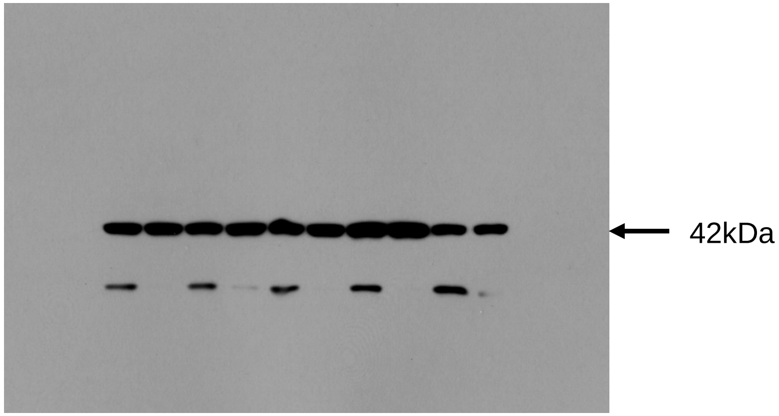

CCR6

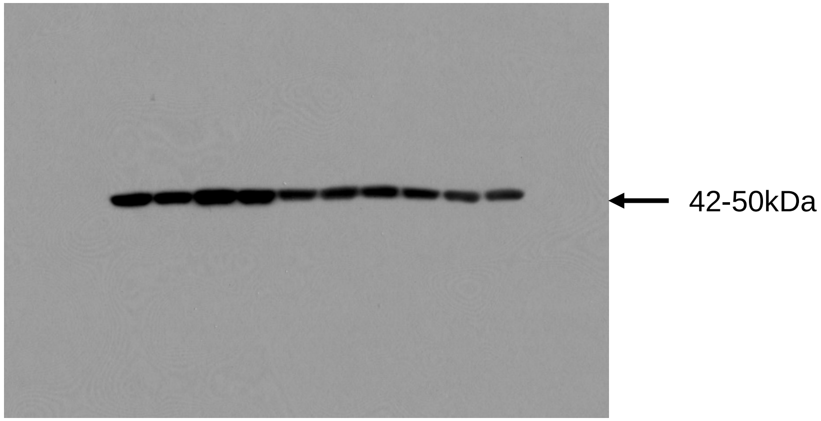

Supplemental Figure 4

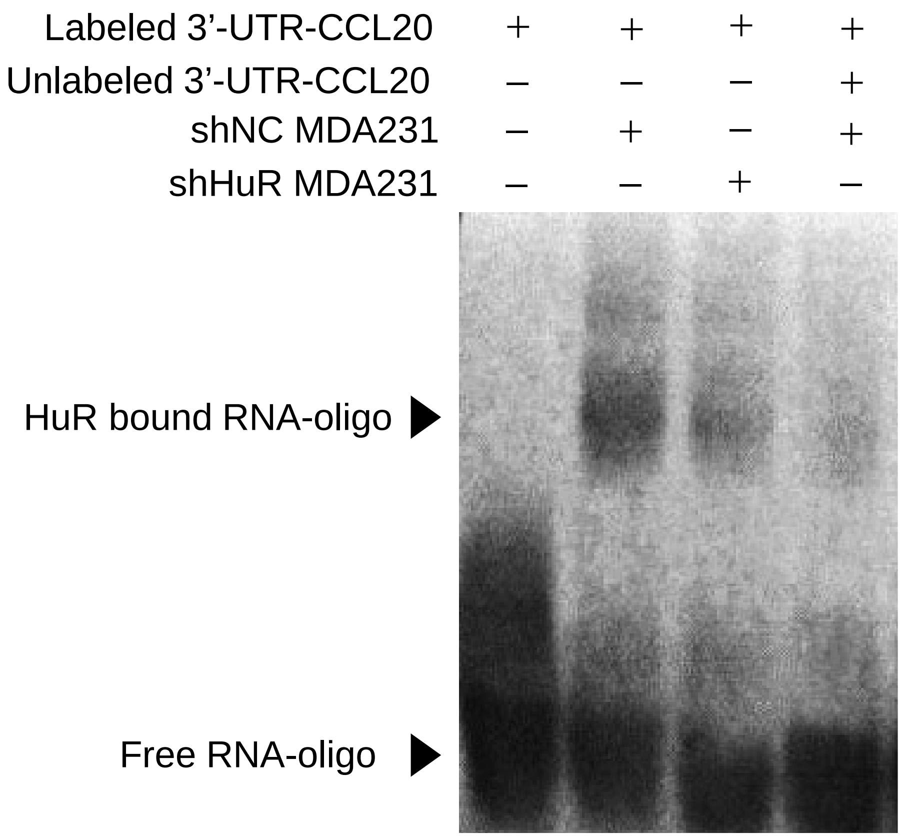

Supplement: Supplementary file 1 — Supplementary information [file 41598_2017_9040_MOESM1_ESM.pdf]
